# User perspectives on the use of X-rays and computer-aided detection for TB

**DOI:** 10.5588/ijtld.22.0232

**Published:** 2022-11-01

**Authors:** R. Barrett, J. Creswell, S. Sahu, Z. Z. Qin

**Affiliations:** Stop TB Partnership, Geneva, Switzerland

Dear Editor,

In 2020, fewer than 60% of the 10 million people with TB were diagnosed and notified.^1^ A global effort is needed to reduce this diagnostic gap and diagnose TB early at the sub-clinical stage.^2^ Chest X-ray (CXR) is a highly sensitive screening tool that can identify early TB disease and improve case detection compared to symptom screening.^2^ Computer-aided detection (CAD) can be used with CXR to circumvent CXR’s high inter- and intra-reader variability, long turnaround times and the lack of radiologists in high-burden countries.^3^–^6^ CAD is therefore particularly beneficial for countries lacking human resources, and in conflict settings, enabling task shifting and automatic reporting.^7^,^8^ Most CAD products generate radiology reports, as well as a continuous numeric abnormality score (0–100 or 0–1) to represent the likelihood that TB-related abnormalities are present, along with a heatmap indicating the location of identified abnormalities. In 2021, the WHO recommended the use of CAD for TB detection in individuals aged ≥15 years, but user experiences are not well documented.^9^ The Stop TB Partnership TB REACH initiative and Digital Health Technology Hub have supported CAD implementation and evaluation for a decade. We therefore conducted a survey (using a self-administered questionnaire) of TB project implementers who use X-ray for TB screening (with or without CAD) from September to October 2021. Both quantitative and qualitative questions were asked. Quantitative questions concerned the use of X-ray (type, reader, turnaround time) and CAD (brand, internet requirement, preferred features). Qualitative questions concerned the advantages and challenges of using CAD, threshold selection and desired features. Participants not using CAD only received questions on CXR, whereas participants using neither system were asked why.

In total, we received 32 responses from 19 countries, with 13 (68%) from high-burden countries (Figure).^10^ Participants were mostly from non-governmental organisations (*n* = 17, 53%), international organisations (*n* = 6, 19%) or health facilities (central and peripheral facilities, and TB clinics) (*n* = 4, 13%). Of the respondents, 19 (59%), were managers and 4 (13%) were clinicians; 27 (84%) participants reported using CXR, while 5 did not. The greatest barriers to accessing CXR were the cost of equipment (60%), cost of human readers (40%) and lack of experienced operators (40%), consistent with previous reports.^11^ The human readers assessing CXRs in all programmes, included radiologists (*n* = 20, 74%), primary physicians (*n* = 11, 41%), pulmonologists (*n* = 11, 41%) and general practitioners (*n* = 10, 37%). Nearly half (*n* = 13, 48%) reported that CXRs were interpreted by one reader, with a median turnaround time of 3 h (interquartile range [IQR] 0.5–24); 41% (*n* = 11) utilised two readers with a shorter median turnaround time of 2 h (IQR 1.0–5.5). Digital radiography was the most frequently used Xray system (*n* = 18, 67%), followed by computed (*n* = 15, 56%) and analogue (*n* = 10, 37%) radiography. Lateral CXR, which CAD cannot read, were received never/rarely (*n* = 7, 29%), sometimes (*n* = 12, 50%), often (*n* = 3, 13%) or all the time (*n* = 2, 8%). Of the 27 respondents using CXR, 19 also used CAD (70%). Of the 8 not using CAD, 7 indicated lack of funding as the principal reason and 3 referred to the cost or incompatible pricing structure of products. Availability of funding was the single factor that would facilitate uptake most for 5 of the 8, and 2 noted that re-training medical officers was the greatest barrier. Among the 19 respondents using CAD, 9 (47%) had 3–12 months’ experience, and a third had 1–5 years’ experience. Two brands of CAD product predominated: qXR (Qure AI,Mumbai, India) was used by 11 participants, and CAD4TB (Delft Imaging, Hertogenbosch, the Netherlands) was used by nine. In total, nine different brands were used. Six programmes worked offline and 11 operated in hybrid mode (involving offline CXR analysis and online results synchronization). Two deployed CAD online-only. The real time availability of results was the greatest benefit of using CAD (*n* = 9, 47%), whereas improved access to services, (in locations without access to specialists), was a primary benefit for eight respondents (42%). The principal advantage depended on the role of the respondent: programme managers highlighted instant CAD results, policymakers cited improved access to radiological assessment in settings without radiologists, and clinicians noted CAD’s high accuracy and expanded ability to detect subclinical or asymptomatic disease. The largest challenges reported were internet availability and bandwidth (*n* = 5, 26%), threshold selection (*n* = 4, 21%) and results interpretation (*n* = 3, 16%), including both misinterpretation of CAD results as diagnostic and lack of trust in CAD results. Two respondents also referred to connectivity challenges between CAD and X-ray software (11%).

**Figure:**
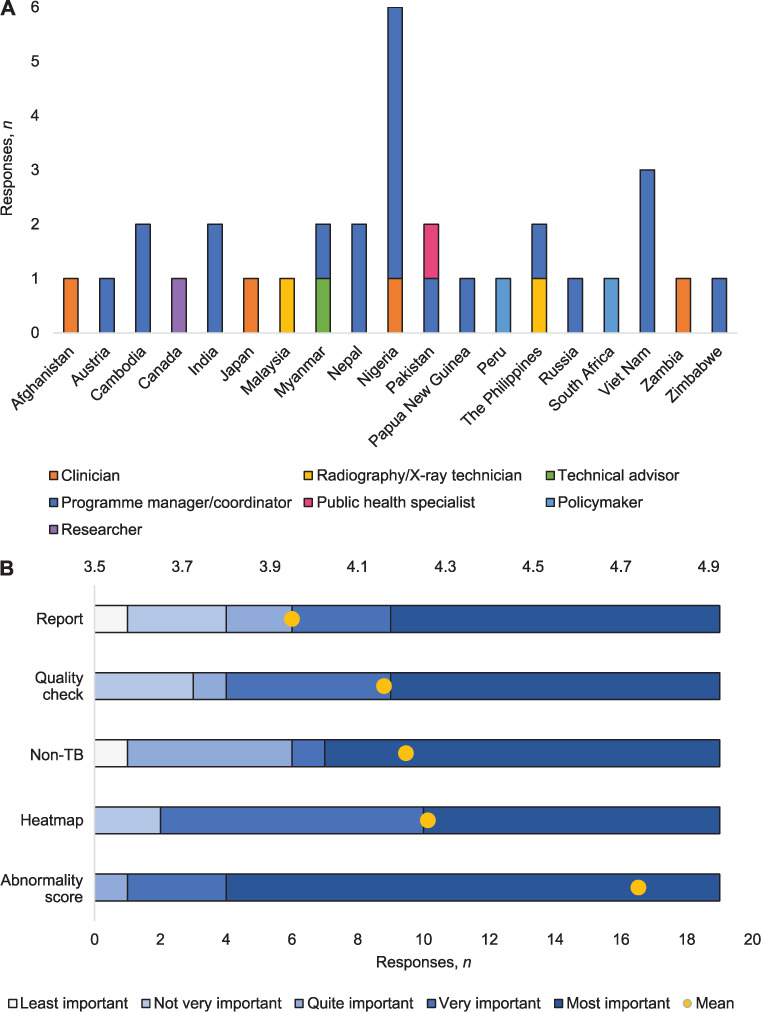
Stacked bar charts highlighting selected results. A) Number of responses by country of work and profession (n = 32). B) How respondents rated five different CAD outputs from least important to most important (n = 19). CAD = computer-aided detection.

The respondents were asked to rate five features of CAD from 1 (least important) to 5 (most important). The 19 respondents generally favoured CAD’s numeric abnormality score (mean 4.74), followed by the heatmap (mean 4.26), the detection of non-TB abnormalities (mean 4.21) and image quality control (mean 4.16) (Figure). Despite being important to end users, the ability of CAD to detect non-TB abnormalities is not independently validated. The automated radiology report, which synthesises results, was considered least important (mean 3.95). Of the 19 respondents, 12 detailed their strategy for selection of a threshold score, above which CXR were classified by CAD as “suggestive of TB” or similar. Over a third (*n* = 5) used a threshold score recommended by the CAD manufacturer, although three adjusted this based on programmatic outcomes or operational research. Other sources of threshold score were operational research (*n* = 4), the national TB programme (*n* = 2, from one site) and peerreviewed publications (*n* = 1). When asked how CAD could be improved, respondents highlighted the ability to identify additional abnormalities (*n* = 4, 21%), read CXR from other angles (e.g., lateral, oblique or apico-lordotic) (*n* = 3, 16%), improved detection of TB in children (*n* = 2, 11%) and older age groups (*n* = 1, 5%).

Despite the limited response rate and the potential bias introduced by only contacting implementers known to the authors, our survey provides insight into experiences of using CAD. Users testified that CAD enabled high throughput, accurate TB screening, with enormously reduced turnaround times, and demonstrated a clear preference for the abnormality score output. Programmes using CAD would benefit from a better understanding of threshold selection and results interpretation, as well as comprehensive evaluation of CAD in children and key populations, and for reading non-TB abnormalities. CAD was limited with regards to the CXR it can process (notably, excluding lateral); concerns that CAD would replace human readers (due to the WHO recommendation) was not reported in this survey. CAD and CXR are promising tools for closing the diagnostic gap, but are not yet accessible to all TB programmes, with lack of funding preventing uptake of both.
